# Sperm culture and bacterial susceptibility to antibiotics in a large andrological population: prevalence and impact on seminal parameters

**DOI:** 10.1007/s10123-022-00273-6

**Published:** 2022-08-24

**Authors:** Soraya Olana, Rossella Mazzilli, Iolanda Santino, Daniela Martinelli, Virginia Zamponi, Manuela Macera, Gerardo Salerno, Fernando Mazzilli, Antongiulio Faggiano, Daniele Gianfrilli

**Affiliations:** 1 Department of Clinical and Molecular Medicine, Sapienza University of Rome, Sant’Andrea University Hospital, Via di Grottarossa, 1036-1039, 00100 Rome, Italy; 2 Department of Neuroscience, Mental Health and Sense Organs, Sapienza University of Rome, Sant’Andrea University Hospital, Rome, Italy; 3 Microbiology Unit, Sant’Andrea University Hospital, Rome, Italy; 4 Dipartimento Di Medicina Clinica E Chirurgia, Sezione Di Endocrinologia, Università Federico II Di Napoli, Naples, Italy; 5 Department of Experimental Medicine, Sapienza University of Rome, Rome, Italy

**Keywords:** Bacterial infections, Male infertility, Semen analysis, Sperm culture, Antibiograms

## Abstract

**Background:**

The aim of this study was to evaluate (i) the prevalence of subjects with a positive sperm culture (SC) for bacteria in subjects with or without genitourinary tract inflammation (GTI); (ii) the actual distribution of the species analysed, according to Gram stain; (iii) the impact on sperm parameters; and (iv) the actual bacterial susceptibility to antibiotics.

**Methods:**

A total of 930 subjects (18–55) years, were retrospectively studied. All the patients underwent SC and in the case of positive tests (CFU > 10^6^), a microbiological susceptibility analysis. The subjects studied were subdivided into group A (*n* = 452), with subjective signs of GTI; group B (*n* = 478), male partners of infertile couples; and group C, 30 healthy normospermic subjects. In group B and in the control group, a semen analysis was performed.

**Results:**

Overall, the prevalence of positive SC was 21.5% (200/930). The prevalence of positive SC in group A (113/200; 56.5%) was significantly higher vs. group B (87/200; 43.5%; *p* = 0.01) and control group (1/30; 3.3%; *p* = 0.0001). In subjects with GTI, the prevalence of asthenozoospermic (96/285; 33.7%) and oligo-asthenozoospermic (98/285; 34.4%) was significantly higher vs. normospermic, oligo-astheno-teratozoospermic, oligozoospermic and azoospermic subjects (22/285 (7.7%), 48/285 (16.8%), 15/285 (5.3%) and 6/285 (2.1%), respectively; *p* = 0.001). Finally, *Enterococcus faecalis* (Gram-positive) and *Escherichia coli* (Gram-negative) showed the highest prevalence of antibiotic resistance.

**Conclusions:**

The prevalence of positive SC is higher in GTI subjects; however, the SC could also be positive in subjects without GTI. Commonly used antibiotics have an increasing risk of being useless for the treatment of bacterial infections. Finally, the diagnosis of GTIs is important also for male fertility.

## Introduction

Over the last years, increasing attention has been paid to genitourinary tract inflammations (GTIs)/infections; several microorganisms can be involved in the pathogenesis of sexually transmitted diseases (STDs), lower urinary tract symptoms (LUTS) and male accessory gland infection (MAGI) (Hannachi et al. 2018; Rodin et al. 2003; Francesco et al. 2011). Various microorganisms have been detected in semen, including chlamydia, mycoplasma, (Fraczek et al. 2016; Dondero et al. 1979), gonococcus, trichomonas and viruses (e.g. HIV, viral hepatitis, HPV) (Fraczek et al. 2016; Vignera et al. 2014), with variable effects on the genital tract, as well as on sperm quality.

Moreover, the most frequent infections are represented by pathogenic bacteria (Pellati et al. 2008). The appropriate microbiological investigations shall include the identification of bacteria and colony-forming units (CFU) by the use of sperm culture (SC). Sometimes, saprophytic bacteria of the genitourinary tract can be found during microbiological investigation (Vignera et al. 2014; Askienazy-Elbhar 2005), and in these cases, it can be difficult to distinguish pathogenic bacteria from saprophytic bacteria. To this regard, Boitrelle et al. and Holm et al. (Boitrelle et al. 2012; Holm et al. 2019) tried to define some thresholds above which treatment should be required in case of a positive SC. Furthermore, an interesting review (Giuliano et al. 2019) described the methods used from 1966 to January 2018 and reviewed the core concepts of interpreting bacterial culture results, including timing of cultures, potential for contamination, interpreting the Gram stain, role of rapid diagnostic tests, conventional antibiotic susceptibility testing and automated testing. On the other hand, accurate information given to the patients regarding the procedure collection should decrease the risk of bacterial contamination, as suggested by WHO guidelines (World Health Organization 2010). However, only samples containing > 10^6^ CFU should be considered positive with microbiological investigations (Dondero et al. 1979; Vignera et al. 2014; Diemer et al. 2003).

The possible correlation between a positive SC and alteration of seminal parameters was also considered (Vignera et al. 2014; Askienazy-Elbhar 2005; Weidner et al. 2013; Vercelloni et al. 1997; Wan et al. 2018; Vilvanathan et al. 2016). In several studies, male GTIs have been associated with 8–35% of male infertility cases (Askienazy-Elbhar 2005). Weidner et al. (Weidner et al. 2013) described a negative impact of infections and inflammation on the parameters of the ejaculate, and MAGI seem to affect 1.6–15% of infertile subjects (Vignera et al. 2014). Regarding the “classic seminal signs of infection/inflammation (WBC increase and hyperviscosity)”, Rodin et al. (Rodin et al. 2003) reported that leucospermia is a poor marker for either bacteriospermia or impaired semen quality. Accordingly, Hannachi et al. (Hannachi et al. 2018) found that the leucocytospermia correlates with bacteriospermia only in 10% of patients.

Regarding the distribution of the species, Vercelloni et al. (n.d.) described a higher prevalence of Gram-positive microorganisms in SCs (as opposed to urinary tract infections), in particular Enterococcus. These data were successively confirmed by Wan et al. (2018), who described that, among sperm donors which presented positive SC, mainly Gram-positive were detected, with a negative impact on sperm parameters. Similarly, Vilvanathan et al. (2016) reported that the prevalence of bacteriospermia in infertile men was 35.3%, of which *Enterococcus faecalis* (30%) was the most common organism isolated, followed by coagulase-negative staphylococci, *Staphylococcus aureus* and *Escherichia coli*; furthermore, *Staphylococcus* species appear to be innocuous, while *Streptococcus viridans* and *E. faecalis* are associated with poorer semen quality and may warrant treatment.

Among the Gram-negative microorganisms, *E. coli* was the most represented (Dondero et al. 1977). De Francesco et al. (2011) reported that the semen culture was positive in 22% of subfertile men; however, samples containing > 10^3^ CFU were also considered positive, mainly *Gardnerella vaginalis*, *E. coli* and *Enterococcus*. Moreover, it was underlined that commonly used antibiotics have an increasing risk of being useless for the treatment of bacterial infections (Reygaert 2018). In fact, the resistance to common antibiotics may not allow for the resolution of bacterial infections. There are numerous mechanisms that favor antibiotic resistance (Reygaert 2018). Nonetheless, few studies have been carried out on microbiological susceptibility in urinary tract infections, and they focused on a single antibiotic or bacterial strain (Holm et al. 2019). To date, few studies have focused on antibiotic resistance in the case of a positive SC. In this regard, Silago et al. described a very high resistance (about 100%) to non-beta lactam antibiotics among Gram-negative bacteria (Silago et al. 2020).

The aim of this study was to evaluate: (*i*) the prevalence of subjects with a positive sperm culture (SC) for pathogenic bacteria in subjects with or without clinical/ultrasound signs of inflammation; (*ii*) the possible impact on sperm parameters; (*iii*) the actual distribution of the species analysed, according to Gram stain; and (*iv*) the actual bacterial susceptibility to antibiotics.

## Materials and methods

### Study population

This retrospective study was conducted in accordance with the principles set forth in the Helsinki Declaration, and in accordance with the “Sapienza” University of Rome Ethics Committee, following standard procedures according to the current guidelines and the ethical standards of the Institutional Research Committee. Written informed consent for participation in future research study was obtained from all participants.

A total of 968 subjects, aged between 18 and 55 years (mean age 37.8 ± 7.8 years), were retrospectively evaluated in this study. These subjects had been referred to our Andrology Unit (Sant’Andrea Hospital — “Sapienza” University of Rome), from January 2016 to November 2020, which referred subjective symptoms of infection/inflammation of the reproductive tract or male partners of infertile couples. Thirty healthy normospermic fertile subjects, who performed semen analysis and SC for andrological screening were included as a control group.

All the patients underwent a full medical history, an andrological physical examination and an ultrasound examination. In all the subjects, SC for common bacteria was carried out. In male partners of infertile couples and in the control group, standard semen analysis was also performed.

The clinical/ultrasound presence of: painful hemiscrotum and testis, increased volume of epididymis, seminal vesicles or prostate, were considered signs of inflammation/infection. All the subjects in the three groups considered presenting these signs were defined as affected by GTI (Lotti and Maggi 2015; Lotti et al. 2021).

Exclusion criteria: (a) bacterial contamination of the sample (two or more CFU from the same sample > 10^4^ and < 10^6^); (b) subjects with signs of urethritis, who needed urethral swabs.

### Semen analysis

The seminal fluid of each participant was collected by masturbation after sexual abstinence between 2 and 7 days. Semen analysis was carried out, according to 2010 WHO guidelines, evaluating spermatozoa morphology, motility and concentration (World Health Organization 2010). The sample container was placed in an incubator (37 °C) for 30–60 min. The physical and chemical characteristics of the seminal liquid were then evaluated (appearance, pH, liquefaction and viscosity). The seminal parameters were then evaluated under the microscope (sperm concentration, percentage of motility and sperm morphology). In particular, Superimposed Image Analysis System (SIAS) software (Delta Sistemi, Rome, Italy), a validated method based on superimposed images, was used to assess sperm motility (Mazzilli et al. 1995). The system can superimpose six sequential frames onto a monitor producing a final image of a complete series of superimposed frames that allows the evaluation of the percentage of motile spermatozoa and their kinetic characteristics.

According to the WHO, we considered: (i) oligozoospermia: total number of spermatozoa below the lower reference limit (15 × 10^6^/mL); ii) (azoospermia: absence of spermatozoa; (iii) asthenozoospermia: percentage of progressively motile spermatozoa below the lower reference limit (32%); and (vi) teratozoospermia: percentage of morphologically normal spermatozoa below the lower reference limit (4%).

### Sperm culture and antibiograms (microbiological susceptibility)

Samples were stirred, after that they were seeded with a sterile loop of 10 µL on a BD Columbia agar with 5% sheep blood (Becton Dikinson, Heidelberg, Germany) and a CHROMagar orientation plate (Becton Dikinson, Heidelberg, Germany). Samples were extracted for each plate, and a semi-quantitative seeding was carried out. Media cultures were incubated at 37 °C for 48 h, which is at an optimal temperature for bacterial growth, after two readings, at 24 and 48 h, were achieved. Among Gram-positive, *E. faecalis*, *Streptococcus agalactiae*, *Staphylococcus haemoliticus*, *Corynebacterium striatum* and *S. aureus* were detected; among Gram-negative, *E. coli*, *Klebsiella pneumoniae*, *Citrobacter koserii*, *Morganella morganii*, *Enterobacter aerogenes aerogenes* and *Proteus mirabilis*.

Samples containing > 10^6^ CFU were considered positive.

Whenever two or more CFU from the same sample > 10^4^ and < 10^6^ were isolated, the sample was considered as bacterial contamination.

In the case of a positive tests (CFU > 10^6^), a microbiological susceptibility analysis was carried out using the DS Phoenix method (manual or semiautomatic), which entails the simultaneous analysis of 100 panels both to identify microorganisms and to perform microbiological susceptibility. These panels can be distinguished based on type of microorganism and antibiogram (EUCAST compliant panels: penicillins: amoxicillin-clavulanic acid, ampicilline, piperacilline, piperacilline-tazobactam; cephalosporin: cefuroxime, cefotaxime, cefepime; beta-lactam: ertapenem, imipenem, meropenem; glycopeptide: teicoplanin, vancomycin; polymyxin: colistin; fluoroquinolones: ciprofloxacin, levofloxacin; aminoglycoside: amikacin, gentamicin, tobramycin; tetracyclines: tetracycline; macrolide: erythromycin; sulfonamide: sulfamethoxazole-trimethoprim; fosfomycin C/G6P, linezolid, tigecycline).

The identification is based on 45–46 biochemical reactions (colourimetric and fluorimetric methods). Antibiogram analysis used two combined systems (optical-colourimetric detection and turbidity reading in microdilution) linked to the growth of the microorganism and to its metabolism, respectively. This method reduces false positives and false negatives and suggests the minimum inhibitory concentration (MIC) in real form.

In this study, microbiological susceptibility analysis was carried out with two panels, according to Gram stain: 14 antibiotics were studied for Gram-positive bacteria, and 19 antibiotics were studied for Gram-negative bacteria. In particular, when a Gram-positive or negative infection occurred, only antibiotics potentially susceptible to both were tested.

### Statistical analysis

Continuous data were described as absolute values, means ± standard deviations (SDs) and ranges. Categorical data were described as absolute values, percentage frequencies and 95% confidence intervals (CIs). We used Fisher’s exact test for significance association between categorical outcome and categorical variables. A *p* < 0.05 was considered statistically significant.

## Results

### Prevalence of subjects with a GTI and positive SC in the groups considered

A total of 968 patients were studied. Of them, 3.9% (38/968) were excluded due to bacterial contamination of the sample, thus leaving a sample of 930 patients.

**Fig. 1:**
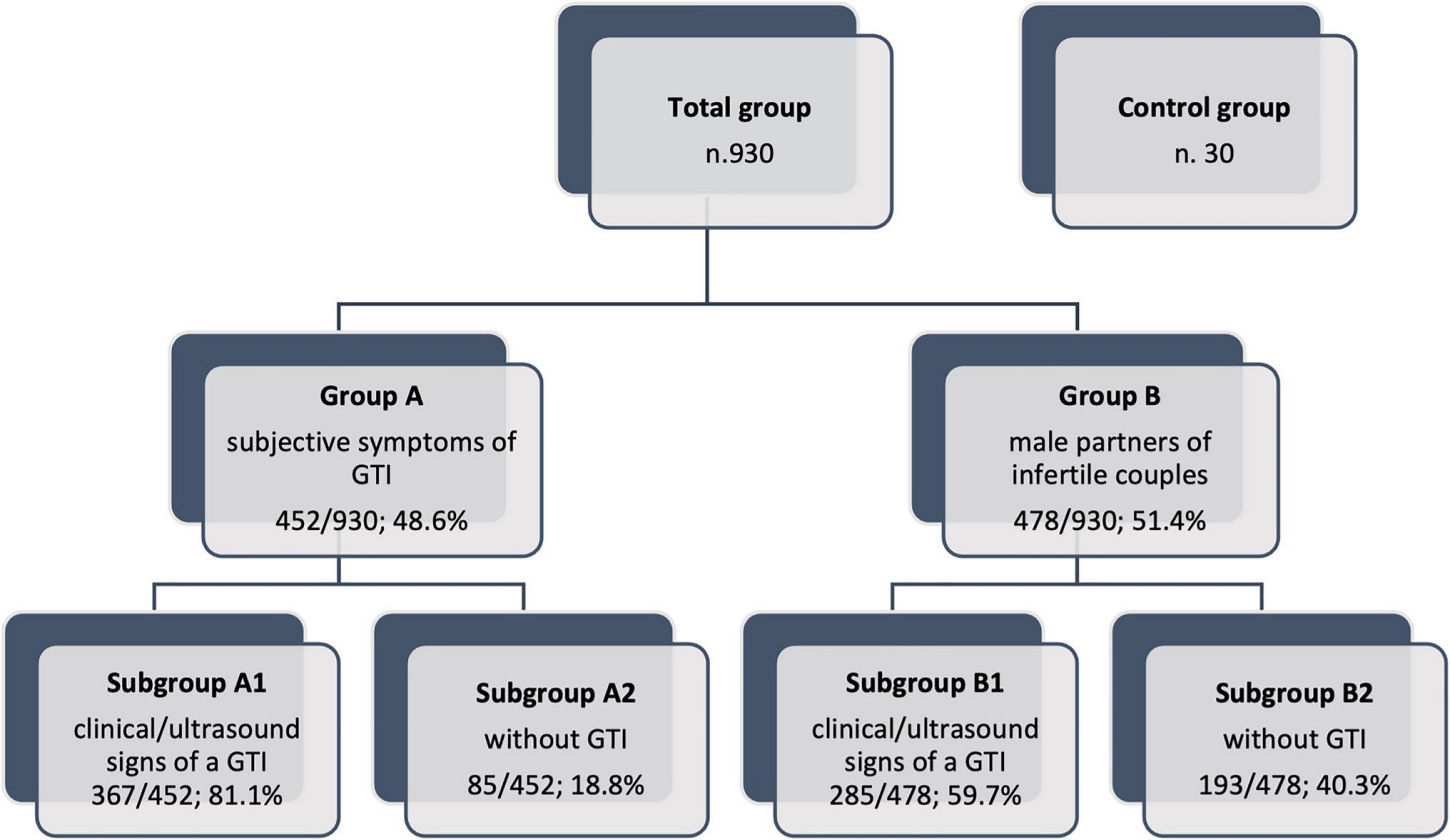
Flow chart of the group’s distribution. *GTI* = *genital tract inflammation*

The subjects were subdivided into: (*i*) group A (452/930 subjects), who referred suspicious symptoms for infection/inflammation of the reproductive tract; (*ii*) group B (478/930 subjects), male partners of infertile couples and (*iii*) group C, 30 healthy normospermic fertile subjects, on whom semen analysis was performed (Fig. 1).

The total prevalence of subjects studied with a positive SC was 21.5% (200/930). The prevalence of subjects with a positive SC in group A (113/452; 25.0%) was significantly higher than those in group B (87/478; 18.2%; *p* = 0.01) as well as in the control group (1/30; 3.3%; *p* = 0.003) (Table 1).

**Table 1:** Prevalence of SC + in the group studied

	n	SC +
Total group	930	200/930 (21.5%)
*Group A*	*452/930 (48.6%)*	*113/452 (25.0%)* ^a b^
*Group B*	*478/930 (51.4%)*	*87/478 (18.2%)*
Control	30	1/30 (3.3%)

*Group A* subjects who referred subjective signs of infections/inflammation of the reproductive tract, *Group B* male partners of infertile couples, *SC* sperm culture

^a^*p* = 0.01 vs. group B

^b^*p* = 0.003 vs. control

Afterwards, according to the presence or absence of signs of GTI, after medical examination, the groups were subdivided, respectively, into subgroup A1 (367/452; 81.1%) and subgroup B1 (285/478; 59.7%) (*p* = 0.001) affected by clinical/ultrasound signs of a GTI, and subgroup A2 (85/452; 18.8%) and subgroup B2 (193/478; 40.3%;) (*p* = 0.001) without signs of a GTI (Table 2; Fig. 1). In group A1, the prevalence of a positive SC was 26.9% (99/367), as compared to that in group B1 (26.2%; 75/285). On the other hand, in subjects not affected by clinical/ultrasound signs of GTI, the prevalence of a positive SC was 16.4% (14/85) in group A2 and 6.2% (12/193) in group B2 (*p* = 0.01).

**Table 2:** Prevalence of SC + according to the presence of clinical/ultrasound signs of genital tract inflammation (GTI) in the subgroups studied

	*n*	SC +	Gram +	Gram −	Gram + / −
*Group A*	*n. 452*	*n. 113/452* *(25.0%) *^*a*^	*n. 46/113* *(40.7%)*	*n. 35/113 (31.0%)*	*n. 32/113 (28.3%)*
Subgroup A1	*n*. 367/452 (81.2%) ^b^	*n*. 99/367 (26.9%)	*n*. 41/99 (41.4%)	*n*. 30/99 (30.3%)	*n*. 28/99 (28.3%)
Subgroup A2	*n*. 85/452 (18.8%) ^c^	*n*. 14/85 (16.4%)	*n*. 5/14 (35.7%)	*n*. 5/14 (35.7%)	*n*. 4/14 (28.6%)
*Group B*	*n. 478*	*n. 87/478* *(18.2%)*	*n. 37/87* *(42.5%)*	*n. 29/87* *(33.4%)*	*n. 21/87* *(24.1%)*
Subgroup B1	285/478 (59.6%)	*n*. 75/285 (26.3%)	*n*. 32/75 (42.7%)	*n*. 25/75 (33.3%)	*n*. 18/75 (24.0)
Subgroup B2	193/478 (40.4%)	*n*. 12/193 (6.2%)	*n*. 5/12 (41.7%)	*n*. 4/12 (33.3%)	*n*. 3/12 (25.0%)
*Control*	*n. 30*	*n. 1/30* *(3.3%)*	*n. 1/1* *(100.0%)*	*–*	*–*
Control 1	1/30 (3.3%)	*n*. 1/1 (100.0%)	*n*. 1/1 (100.0%)	–	–
Control 2	29/30 (96.7%)	*n*. 0/29 (–)	–	–	–

*Subgroups A1, B1 and control 1* subjects affected by GTI, *subgroup A2, B2 and control 2* subjects without GTI

^a^*p* = 0.012 vs. group B

^b^*p* = 0.001 vs. group B1

^c^*p* = 0.001 vs. group B2

### Sperm parameters

The subjects of subgroup B1 and subgroup B2 were clustered, according to the sperm parameters, into six groups: (a) OAT, (b) oligozoospermic, (c) asthenozoospermic, (d) oligoasthenozoospermic, (e) azoospermic, and (f) normospermic (Table 3); no significant differences in age were observed among the subgroups. In subjects with GTI (subgroup B1), the prevalence of asthenozoospermic and oligoasthenozoospermic subjects was significantly higher compared to normospermic, OAT, oligozoospermic and azoospermic subjects (96/285 (33.7%) and 98/285 (34.4%) vs. 22/285 (7.7%), 48/285 (16.8%), 15/285 (5.3%) and 6/285 (2.1%), respectively; *p* = 0.001).

**Table 3:** Prevalence of SC + in subgroup B1 (affected by GTI) and in subgroup B2 (without GTI) according to the seminal parameters

	*n*	SC +	Gram +	Gram −	Gram + / −
*Subgroup B1*	*n. 285*	*n. 75/285* *(26.3%)*	*n. 32/75* *(42.7%)*	*n. 25/75* *(33.3%)*	*n. 18/75* *(24.0)*
OAT	*n*. 48/285 (16.8%)	13/48 (27.0%)	*n*. 5/13 (38.5%)	*n*. 4/13 (30.8%)	*n*. 4/13 (30.8%)
Oligospermic	*n*. 15/285 (5.3%)	3/15 (20.0%)	*n*. 2/3 (66.7%)	*n*. 0/3 (–)	*n*. 1/3 (33.3%)
Asthenospermic	*n*. 96/285 (33.7%) ^a^	36/96 (37.5%)^c^	16/36 (44.4%)	13/36 (36.1%)	7/36 (19.4%)
Oligoasthenospermic	*n*. 98/285 (34.4%) ^b^	19/98 (19.3%)	*n*. 8/19 (42.1%)	*n*. 5/19 (26.3%)	*n*. 6/19 (31.6%)
Azoospermic	*n*. 6/285 (2.1%)	1/6 (16.7%)	*n*. 0/1 (–)	*n*. 1/1 (100.0%)	*n*. 0/1 (–)
Normospermic	*n*. 22/285 (7.7%)	*n*. 3/22 (22.7%)	*n*. 2/3 (66.7%)	*n*. 1/3 (33.3%)	*n*. 0/3 (–)
*Subgroup B2*	*n. 193*	*n. 12/193* *(6.2%)*	*n. 5/12* *(41.7%)*	*n. 4/12* *(33.3%)*	*n. 3/12* *(25.0%)*
OAT	*n*. 13/193 (6.7%)	1/13 (7.6%)	*n*. 0/1 (–)	*n*. 1/1 (100.0%)	*n*. 0/1 (–)
Oligospermic	*n*. 16/193 (8.3%)	0/16 (–)	(–)	(–)	(–)
Asthenospermic	*n*. 22/193 (11.4%)	6/22 (27.2%)e	*n*. 3/6 (50.0%)	*n*. 1/6 (16.7%)	*n*. 2/6 (33.3%)
Oligoasthenospermic	*n*. 24/193 (12.4%)	4/24 (24.3%)	*n*. 2/4 (50.0%)	*n*. 1/4 (25.0%)	*n*. 1/4 (25.0%)
Azoospermic	*n*. 6/193 (3.1%)	0/6 (–)	(–)	(–)	(–)
Normospermic	*n*. 112/193 (58.1%) ^d^	1/112 (0.9%)	*n*. 0/1 (–)	*n*. 1/1 (100.0%)	*n*. 0/1 (–)

*OAT* oligoasthenozoospermic

^a^*p* = 0.001 vs. normospermic, oligozoospermic, OAT and azoospermic Subgroup B1

^b^*p* = 0.001 vs. normospermic, oligozoospermic, OAT and azoospermic subgroup B1

^c^*p* = 0.04 vs. normospermic subgroup B1

^d^*p* = 0.0001 vs. all subgroup B2

^e^*p* = 0.0001 vs. normospermic vs. subgroup B2

On the contrary, in subgroup B2, the prevalence of normospermic subjects was significantly higher compared to the other subgroups (112/193 (58.1%) vs. 13/193 (6.7%), 16/193 (8.3%), 22/193 (11.4%), 24/193 (12.4%), and 6/193 (3.1%) in OAT, oligozoospermic, asthenozoospermic, oligoasthenozoospermic and azoospermic subgroups, respectively; *p* = 0.001).

Among subgroup B1, the prevalence of a positive SC was higher in the asthenozoospermic subgroup compared to OAT, oligozoospermic, azoospermic and normospermic subgroups (36/96 (37.5%) vs. 13/48 (27.0%), 3/15 (20.0%), 19/98 (19.3%), 1/6 (16.7%) and 3/22 (22.7%), respectively; *p* = 0.01). No significant differences were observed among Gram-positive or Gram-negative microorganism prevalence.

Among subgroup B2, the prevalence of a positive SC in the asthenoszoopermic subgroup was higher compared to the normospermic subgroup (6/22 (27.2%) vs. 1/112 (0.9%); *p* = 0.0001). No significant differences were observed among Gram-positive or Gram-negative microorganism prevalence.

Finally, the seminal signs of infection/inflammation (WBC increase and semen hyperviscosity (SHV)) were analysed (Table 4). Considering subgroup B1, the prevalence of SHV, WBC increase, or both was comparable (21/285 (7.4%), 28/285 (9.8%) and 26/285 (9.1%), respectively; *p* = n.s.); considering subgroup B2, the prevalence of subjects with both SHV and WBC increase was significantly lower compared to SHV alone or WBC increase (5/193 (2.6%) vs. 20/193 (10.4%) and 16/193 (8.3%), respectively; *p* = 0.03).

**Table 4:** Prevalence of SC + in subgroups B1 (affected by GTI) and in subgroups B2 (without GTI) according to the seminal5 semen viscosity and WBC parameters

	*n*	SC +	Gram +	Gram −	Gram + / −
*Subgroup B1*	*n. 285*	*n. 75/285* *(26.3%)*	*n. 32/75* *(42.7%)*	*n. 25/75* *(33.3%)*	*n. 18/75* *(24.0)*
SHV increase	*n*. 21/285 (7.4%)	10/21 (47.7%)^a,b^	*n*. 4/10 (40.0%)	*n*. 4/10 (40.0%)	*n*. 2/10 (20.0%)
WBC increase	*n*. 28/285 (9.8%)	9/28 (32.1%)	*n*. 4/9 (44.5%)	*n*. 3/9 (33.3%)	*n.* 2/9 (22.2%)
SHV/WBC increase	*n*. 26/285 (9.1%)	12/26 (46.1%)	*n*. 5/12 (41.7%)	*n*. 4/12 (33.3%)	*n*. 3/12 (25.0%)
No increase	*n*. 210/285 (73.7%)	44/210 (20.9%)	*n*. 19/44 (43.2%)	*n*. 14/44 (31.8%)	*n*. 11/44 (25.0%)
*Subgroup B2*	*n. 193*	*n. 12/193* *(6.2%)*	*n. 5/12* *(41.7%)*	*n. 4/12* *(33.3%)*	*n. 3/12* *(25.0%)*
SHV increase	*n*. 20/193 (10.4%)	3/20 (15.0%)	*n*. 2/3 (66.7%)	*n*. 0/3 (–)	*n*. 1/3 (33.3%)
WBC increase	*n*. 16/193 (8.3%)	5/16 (31.2%)	*n*. 2/5 (40.0%)	*n*. 2/5 (40.0%)	*n*. 1/5 (20.0%)
SHV/WBC increase	*n*. 5/193 (2.6%)^c^	2/5 (40.0%)	*n*. 1/2 (50.0%)	*n*. 1/2 (50.0%)	*n*. 0/2 (–)
No increase	*n*. 152/193 (78.7%)	2/152 (1.3%)	*n*. 0/2 (–)	*n*. 1/2 (50.0%)	*n*. 1/2 (50.0%)

*SHV* sperm hyper viscosity, *WBC* white blood cell

^a^*p* = 0.001 vs. subgroup B2

^b^*p* = 0.002 vs. no increase subgroup B1

^c^*p* = 0.02 vs. no SHV and WBC

Comparing subgroup B1 and B2, the prevalence of SHV increase was significantly higher in the first one (10/21 (47.7%) vs. 3/20 (15.0%); *p* = 0.001). No significant differences were observed among Gram-positive or Gram-negative microorganism prevalence.

### Distributions of the species

The distributions of the species analysed, according to Gram stain, are shown in Table 5. The prevalence of Gram-positive species was higher in both group A and group B, as compared with that for the Gram-negative or Gram-positive/Gram-negative species; however, this finding did not reach statistical significance (46/113 (40.7%) and 37/87 (42.5%) vs. 35/113 (31.0%) and 29/87 (33.4%), respectively; *p* = 0.2). Furthermore, considering the control group, the only Gram-positive species were detected (2/2, 100%).

**Table 5:** Distribution of the Gram + Gram − and Gram + / − species in the groups considered

	Group A	Group B	Control
	*n*. 113/452 (25.0%)	*n*. 87/478 (18.2%)	*n*. 2/30 (6.7%)
Gram +	*n*. 46/113 (40.7%)	*n*. 37/87 (42.5%)	*n*. 2/2 (100.0%)
*E. faecalis*	*n*. 23/46 (50.0%) ^a^	*n*. 23/37 (62.2%) ^a^	*n*. 1/2 (50.0%)
*S. agalactiae*	*n*. 12/46 (26.1%) ^b^	*n*. 8/37 (21.6%) ^b^	*n*. 0/2 (–)
*S. haemoliticus*	*n*. 6/46 (13.0%)	*n*. 2/37 (5.4%)	*n*. 0/2 (–)
*C. striatum*	*n*. 2/46 (4.4%)	*n*. 2/37 (5.4%)	*n*. 0/2 (–)
*S. haemoliticus–E. faecalis*	*n*. 2/46 (4.4%)	*n*. 1/37 (2.7%)	*n*. 0/2 (–)
*S. aureus*	*n*. 1/46 (2.1%)	*n*. 1/37 (2.7%)	*n*. 1/2 (50.0%)
Gram −	*n*. 35/113 (31.0%)	*n*. 29/87 (33.4%)	*n*. 0/30 (–)
*E. coli*	*n*. 22/35 (62.8%) ^c^	*n*. 19/29 (65.6%) ^c^	(–)
*K. pneumoniae*	*n*. 3/35 (8.7%)	*n*. 5/29 (17.3%)	(–)
*C. koserii*	*n*. 2/35 (5.7%)	*n*. 2/29 (6.9%)	(–)
*M. morganii*	*n*. 1/35 (2.8%)	*n*. 1/29 (3.4%)	(–)
*E. aerogenes*	*n*. 3/35 (8.7%)	*n*. 0/29 (–)	(–)
*E. coli–E. aerogenes*	*n*. 2/35 (5.7%)	*n*. 1/29 (3.4%)	(–)
*E. coli–K. pneumoniae*	*n*. 1/35 (2.8%)	*n*. 1/29 (3.4%)	(–)
*P. mirabilis*	*n*. 1/35 (2.8%)	*n*. 0/29 (–)	(–)
Gram + / −	*n*. 32/113 (28.3%)	*n*. 21/87 (24.1%)	*n*. 0/30 (–)
*E. faecalis—E. coli*	*n*. 15/32 (46.9%)	*n*. 11/21 (52.4%)	(–)
*E. faecalis–M. morganii*	*n*. 12/32 (37.5%)	*n*. 4/21 (19.0%)	(–)
*E. faecalis–C. koserii*	*n*. 1/32 (3.1%)	*n*. 2/21 (9.5%)	(–)
*S. aureus–A. baumanii*	*n*. 1/32 (3.1%)	*n*. 0/21 (–)	(–)
*E. faecalis–P. mirabilis*	*n*. 2/32 (6.3%)	*n*. 2/21 (9.5%)	(–)
*E. faecalis–K. pneumoniae*	*n*. 0/32 (–)	*n*. 1/21 (4.8%)	(–)
*E. coli–G. vaginalis*	*n*. 1/32 (3.1%)	*n*. 1/21 (4.8%)	(–)

^a,b,c^*p* = 0.01 vs. other species

Among the patients with Gram-positive infections, the most frequent species observed were *E. faecalis* and *S. agalactiae* both in group A (23/46 (50.0%) and 12/46 (26.1%); *p* = 0.01 vs. other species) and group B (23/37 (62.2%) and 8/37 (21.6%); *p* = 0.01 vs. other species), while among the subjects with Gram-negative infections, the most frequent species was *E. coli* both in group A (22/35 (62.8%); *p* = 0.01 vs. other species) and in group B (19/29 (65.5%); *p* = 0.01 vs. other species).

The prevalence of these bacteria was confirmed in the cases of mixed infection (Gram-positive and Gram-negative (*E. faecalis* plus *E. coli* 15/32 (46.9%) in group A and 11/21 (52.4%) in group B; *p* < 0.01 vs. other combined infections). There were no mixed infections caused by two different Gram-negative bacteria at the same time, nor were there any caused by two different Gram-positive bacteria.

### Antibiotic resistance

The distributions of resistance of the species analysed, according to Gram stain, are shown in Table. 6. Among the samples positive for Gram-positive microorganisms, the antibiotics with high resistance were tetracycline (28/83, 33.7%) and sulfamethoxazole-trimethoprim (39/83, 46.9%), while among the samples positive for Gram-negative microorganisms, the antibiotics with high resistance were two antibiotics from the penicillin group (ampicillin 33/64, 51.5% and piperacillin 28/64, 43.5%). Furthermore, also amoxicillin-clavulanic acid (16/64, 25.0%) and fluoroquinolones (ciprofloxacin 15/64, 23.4% and levofloxacin 13/64, 20.3%) showed increased resistance to Gram-negative bacteria eradication. In the occurrence of a mixed infection (Gram-positive and Gram-negative), bacteria showed susceptibility to the same antibiotics as in the case of single infection, but they were less effective than in single infections (fosfomycin 22/53, 41.5%), with others being almost completely ineffective (e.g., sulfamethoxazole-trimethoprim 42/53, 79.2%).

**Table 6:** Microbiological susceptibility panels of main antibiotics used to bacterial infections of genitourinary tract

Antibiotics		*Gram* +	*Gram − *	*Gram* + */ − *
		*n*. 83	*n*. 64	*n*. 53
Penicillins	Amoxicillin-Clavulanic acid	*n*. 4/83 (4.0%)	*n*. 16/64 (25.0%)	*n*. 21/53 (39.6%)
	Ampicillin	*n*. 4/83 (4.0%)	*n*. 33/64 (51.5%)	*n*. 39/53 (73.6%)
	Piperacilline	-	*n*. 28/64 (43.5%)	-
	Piperacilline-Tazobactam	-	*n*. 0/64 (0%)	-
Cephalosporin	Cefuroxime	-	*n*. 8/64 (12.5%)	-
	Cefotaxime	-	*n*. 10/64 (15.6%)	-
	Cefepime	-	*n*. 7/64 (10.9%)	-
Beta-Lactam	Ertapenem	-	*n*. 0/64 (0%)	-
	Imipenem	*n*. 0/83 (0%)	*n*. 1/64 (1.5%)	-
	Meropenem	-	*n*. 0/64 (0%)	-
Glycopeptide	Teicoplanin	*n*. 0/83 (0%)	-	-
	Vancomycin	*n*. 5/83 (6.0%)	-	-
Polymyxin	Colistin	-	*n*. 7/64 (10.9%)	-
Fluoroquinolones	Ciprofloxacin	*n*. 7/83 (8.4%)	*n*. 15/64 (23.4%)	*n*. 4/53 (7.5%)
	Levofloxacin	*n*. 2/83 (2.4%)	*n*. 13/64 (20.3%)	*n*. 3/53 (5.6%)
Aminoglycoside	Amikacin	-	*n*. 1/64 (1.5%)	-
	Gentamicin	*n*. 1/83 (1.2%)	*n*. 7/64 (10.9%)	*n*. 4/53 (7.5%)
	Tobramycin	-	*n*. 4/64 (6.2%)	-
Tetracyclines	Tetracycline	*n*. 28/83 (33.7%)	-	-
Macrolide	Erythromycin	*n*. 14/83 (16.8%)	-	-
Sulfonamide	Sulfamethoxazole-trimethoprim	*n*. 39/83 (46.9%)	*n*. 6/64 (9.3%)	*n*. 42/53 (79.2%)
	Fosfomycin C/G6P	*n*. 1/83 (1.2%)	*n*. 4/64 (6.2%)	*n*. 22/53 (41.5%)
	Linezolid	*n*. 0/83 (0%)		-
	Tigecycline	*n*. 4/83 (4.8%)	*n*.12/64 (18.8%)	*n*. 18/53 (34.0%)

Regarding antibiotic resistance trends in bacterial strains, *E. faecalis* (Gram-positive) and *E. coli* (Gram-negative) showed the highest prevalence of antibiotic resistance. In particular, *E. faecalis* showed antibiotic resistance for sulfamethoxazole-trimethoprim; likewise, *E. coli* showed antibiotic resistance for amoxicillin-clavulanic acid and ciprofloxacin.

## Discussion

Bacterial infections are a frequent reason for an andrological consultation. A microbiological investigation is important for complete diagnostic management, and therefore, samples must observe criteria of suitability (Fraczek et al. 2016; Vignera et al. 2014; Calogero et al. 2017). However, SC can be reliable if patients followed the right procedure for sample collection, as suggested by WHO guidelines (World Health Organization 2010), in order to decrease the risk of bacterial contamination, since more than 7% of the samples could be tainted. Nonetheless, only samples containing > 10^6^ CFU should be considered positive in microbiological investigations.

In this work, the prevalence of a positive SC in a large andrological population was evaluated. The two groups studied (group A, characterized by subjects who referred symptoms suspicious for infection/inflammation of the genitourinary tract, and group B, characterized by male partners of infertile couples) were divided into subgroups according to the presence of clinical/ultrasound signs of GTI (A1 and B1) or the absence of clinical signs (A2 and B2), after medical examination. Interestingly, in subgroups A1 and B1, the prevalence of a positive SC was comparable, around 26%. Several studies explained the presence of a positive SC in subjects without signs or symptoms of a GTI as the contamination of semen (Rybar et al. 2012). In our study, the tainted samples were excluded, so we can speculate that these were very early stages of infections; therefore, there was the opportunity to treat at an early stage or asymptomatic bacteriospemia. To this regard, Esfandiari et al. (2002) highlighted that a lack of correlation between positive SCs and sperm characteristics may be indicative of early or mild/subclinical infection.

Regarding the correlation between inflammation of the male reproductive system and infertility, a strong association has been reported (Vignera et al. 2014, 2019). Semen quality can be altered by the inflammatory process, anatomical obstruction, presence of an unsuitable microenvironment due to oxidative stress and/or spermatogenesis dysregulation (Aitken et al. 1989; Aydemir et al. 2008). Bacteria can also participate in intrinsic mitochondria-dependent apoptotic cell death mechanisms (Fraczek et al. 2016). According to ultrasound, infertile patients with hypertrophic-congestive MAGI have a better sperm quality compared with fibro-sclerotic MAGI (Vignera et al. 2011).

In our study, an interesting observation was that the presence of a GTI seemed to affect mainly the motility of sperm (i.e. asthenozoospermic, oligoasthenozoospermic and OAT groups). This may be due to the fact that the damage is greater in the sites where the spermatozoa live longer and where they acquired competent motility, namely the epididymis. Interstitial oedema, loss of epithelial integrity and elevated levels of pro-inflammatory cytokines may induce impaired semen motility (Fijak et al. 2018).

In addition, some inflammatory parameters may be present in the seminal fluid, such as the increase in WBCs and SHV. An interesting finding is that, while in subjects with a WBC increase the prevalence of a positive SC was comparable in groups B1 and B2, and in subjects with SHV, the prevalence of a positive SC was highly significant in group B1 (affected by a GTI), as compared to group B2. This seems to confirm the hypothesis of a genetic nature in the determinism of SHV (Rossi et al. 2004; Elia et al. 2009).

Our study showed some significant variations in the prevalence of bacterial positivity of the male genital tract compared to previous studies, both in infertile patients than in subjects screened for inflammation/infection. According to Vercelloni et al. (n.d.), we observed a higher prevalence of Gram-positive compared to Gram-negative infections. Similarly, among the subjects positive for Gram-positive microorganisms, the most frequent species observed was *E. faecalis*, but there was also a significant increase of *S. agalactiae*. On the other hand, among the subjects positive for Gram-negative microorganisms, the most frequent species was *E. coli*; furthermore, *Proteus mirabilis* was less frequent than in the previous study.

Lastly, we evaluated the microbiological resistance to common antibiotics used to treat bacterial infections of the genital tract. Regarding Gram-positive strains, resistance to several antibiotics (i.e. ciprofloxacin and sulfamethoxazole-trimethoprim) was highlighted. Resistance to penicillin and fluoroquinolones was observed among Gram-negative microorganisms (amoxicillin-clavulanic acid, ampicillin, piperacillin and ciprofloxacin). Therefore, there is high antibiotic resistance, which can impair the effectiveness of these agents. This could be due to (a) a greater ability of the bacterium to develop “escape mechanisms”; (b) a different attitude of the bacterium to become resistant based on the district in which the infection is generated; or (c) a different predisposition of the individual to develop such resistances (Reygaert 2018). Comparing our data with previous studies, we found discordant results; specifically, Silago et al. highlighted a higher percentage of resistance among Gram-negative bacteria to antibiotics ampicillin, trimethoprim-sulphamethoxazole and amoxycillin-clavulanic acid (100%, 100% and 92.1%, respectively) (Silago et al. 2020). The difference between these results could be related to antibiotic consumption as well as to selection of patients.

However, the interest in the literature for antibiotic resistance has mainly concerned the urinary tract; therefore, the majority of data refer to urine samples (Holm et al. 2019; Giuliano et al. 2019; Weidner et al. 2013).

The main limitation of this study is its retrospective nature and the limited sample concerning single microorganisms, as well as the absence of data concerning urethral swabs. Furthermore, we could not use the term male adnexal glands infection (“MAGI”), because in the latter, the characterization is given by the presence of oligozoospermia, asthenozoospermia or teratozoospermia, in addition to the clinical parameters, ultrasound and seminal and microbiological signs. In this study, however, the possible correlation between GTIs and seminal parameters was studied.

## Conclusion

In conclusion, the prevalence of a positive SC was higher in subjects presenting with signs of a GTI; however, it could also be positive in subjects without any signs of a GTI and should be taken into consideration. The study also showed some variations in the prevalence of bacterial positivity of the male genital tract compared to previous studies. Commonly used antibiotics have an increasing risk of being useless for the treatment of future bacterial infections. Finally, these investigations are also useful in the management of infertility; therefore, the diagnosis of male GTIs is essential not only for treatment of symptomatic conditions but also for the impact on male fertility.

## References

[CR1] Aitken RJ, Clarkson JS, Hargreave TB (1989). Analysis of the relationship between defective sperm function and the generation of reactive oxygen species in cases of oligozoospermia. J Androl.

[CR2] Askienazy-Elbhar M (2005). Male genital tract infection: the point of view of the bacteriologist. Gynecol Obstet Fertil.

[CR3] Aydemir B, Onaran I, Kiziler AR (2008). The influence of oxidative damage on viscosity of seminal fluid in infertile men. J Androl.

[CR4] Boitrelle F, Robin G, Lefebvre C (2012). Bacteriospermia in assisted reproductive techniques: effects of bacteria on spermatozoa and seminal plasma, diagnosis and treatment. Gynecol Obstet Fertil.

[CR5] Calogero AE, Duca Y, Condorelli RA (2017). Male accessory gland inflammation, infertility, and sexual dysfunctions: a practical approach to diagnosis and therapy. Andrology.

[CR6] De Francesco MA, Negrini R, Ravizzola G (2011). Bacterial species presents in the lower male genital tract: a five-year retrospective study. Eur J Contracept Reprod Health Care.

[CR7] Diemer T, Huwe P, Ludwig M (2003). Urogenital infection and sperm motility. Andrologia.

[CR8] Dondero F, Giovenco P, Mazzilli F (1977). Seminologic features in infertile patients with positive sperm cultures. Minerva Urol Nov-Dec.

[CR9] Dondero F, Giovenco P, Mazzilli F (1979). Seminological aspects in infertile patients with mycoplasm infection. Minerva Urol Jan-Mar.

[CR10] Elia J, Delfino M, Imbrogno N (2009). Human semen hyperviscosity: prevalence, pathogenesis and therapeutic aspects. Asian J Androl.

[CR11] Esfandiari N, Saleh RA, Abdoos M, Rouzrokh A, Nazemian Z (2002). Positive bacterial culture of semen from infertile men with asymptomatic leukocytospermia. Int J Fertil Womens Med.

[CR12] Fijak M, Pilatz A, Hedger MP, Nicolas N, Bhushan S, Michel V, Tung KSK, Schuppe HC, Meinhardt A (2018). Infectious, inflammatory and ‘autoimmune’ male factor infertility: how do rodent models inform clinical practice?. Hum Reprod Update.

[CR13] Fraczek M, Hryhorowicz M, Gill K (2016). The effect of bacteriospermia and leukocytospermia on conventional and nonconventional semen parameters in healthy young normozoospermic males. J Reprod Immunol.

[CR14] Giuliano C, Patel CR, Kale-Pradhan PB (2019). A guide to bacterial culture identification and results interpretation. P T.

[CR15] Hannachi H, Elloumi H, Hamdoun M (2018). Bacteriospermia: effects on semen parameters. Gynecol Obstet Fertil Senol.

[CR16] Holm A, Cordoba G, Aabenhus R (2019). Prescription of antibiotics for urinary tract infection in general practice in Denmark. Scand J Prim Health Care.

[CR17] La Vignera S, Vicari E, Condorelli R (2011). Hypertrophic-congestive and fibro-sclerotic ultrasound variants of male accessory gland infection have different sperm output. J Endocrinol Invest.

[CR18] La Vignera S, Condorelli RA, Vicari E (2014). Microbiological investigation in male infertility: a practical overview. J Med Microbiol.

[CR19] La Vignera S, Vicari E, Condorelli RA, Cannarella R (2019). Urogenital infections in patients with diabetes mellitus: beyond the conventional aspects. Int J Immunopathol Pharmacol.

[CR20] Lotti F, Maggi M (2015). Ultrasound of the male genital tract in relation to male reproductive health. Hum Reprod Update.

[CR21] Lotti F, Frizza F, Balercia G (2021). The European Academy of Andrology (EAA) ultrasound study on healthy, fertile men: scrotal ultrasound reference ranges and associations with clinical, seminal, and biochemical characteristics. Andrology.

[CR22] Mazzilli F, Rossi T, Sabatini L, Dondero F (1995). Superimposed image analysis system (SIAS) software: a new approach to sperm motility assessment. Fertil Steril.

[CR23] Pellati D, Mylonakis I, Bertoloni G, Fiore C, Andrisani A, Ambrosini G, Armanini D (2008). Genital tract infections and infertility. Eur J Obstet Gynecol Reprod Biol.

[CR24] Reygaert WC (2018). An overview of the antimicrobial resistance mechanisms of bacteria. AIMS Microbiol.

[CR25] Rodin DM, Larone D, Goldstein M (2003). Relationship between semen culture, leukospermia and semen analysis in men undergoing fertility evaluation. Fertil Steril.

[CR26] Rossi T, Grandoni F, Mazzilli F (2004). High frequency of (TG)mTn variant tracts in the cystic fibrosis transmembrane conductance regulator gene in men with high semen viscosity. Fertil Steril.

[CR27] Rybar R, Prinosilova P, Kopecka V (2012). The effect of bacterial contamination of semen on sperm chromatin integrity and standard semen parameters in men from infertile couples. Andrologia.

[CR28] Silago V, Mukama Y, Haule AL, Chacha F, Igenge J, Mushi MF, Mshana SE (2020). Bacteriospermia, extended spectrum beta lactamase producing Gram-negative bacteria and other factors associated with male infertility in Mwanza, Tanzania: a need of diagnostic bacteriology for management of male infertility. Afr Health Sci.

[CR29] Vercelloni B, Sappa M, La Scala M (1977) La spermiocoltura nella infertilità maschile; Estratto da Il Laboratorio di Patologia Clinica n.2–1977

[CR30] Vilvanathan S, Kandasamy B, Jayachandran AL (2016). Bacteriospermia and its impact on basic semen parameters among infertile men. Interdiscip Perspect Infect Dis.

[CR31] Wan L, Chen L, Huang J (2018). Bacterial culture of donor semen: analysis of results. Zhonghua Nan Ke Xue.

[CR32] Weidner W, Pilatz A, Diemer T (2013). Male urogenital infections: impact of infection and inflammation on ejaculate parameters. World J Urol.

[CR33] World Health Organization (2010). Laboratory manual for the examination and processing of human semen.

